# Miscellaneous

**Published:** 1855-04

**Authors:** 


*Changes Editorial.*—Dr. G. S. Jones has retired from the post of Associate Editor of the Boston Medical Journal, and Drs. Wm. W. Morland and Francis Minot, have taken his place.

Dr. J. O. Hill, has commenced the publication of the "Medical Councillor," a new Journal of 16 pages issued weekly, at Columbus, Ohio.

Drs. D. J. Cain and F. P. Porcher of Charleston, S. C., have retired from the editorships of that excellent periodical, the Charleston Medical Journal and Review, and has been succeeded by Dr. C. Happoldt.

Dr. Bennett Dowler, of New Orleans, has announced his retirement from the New Orleans Medical and Surgical Journal, and his intention to commence the publication on the first of May next, of a "New Orleans Quarterly Journal of Medicine."

Each number of the new Quarterly is to contain two hundred and sixteen pages octavo.

*Changes Professorial.*—Dr. E. Brown Sequard, late of Paris, has commenced his duties as Professor of Physiology in the College of Richmond, Va. Professors Walter Channing and Jacob Bigelow, two of the oldest members of the Faculty of the Medical Department of Harvard University at Boston, have retired from the Faculty of that time-honored institution, and have been succeeded by the appointment of Dr. D. H. Storer to the chair of Obstetrics and Medical Jurisprudence, and Dr. Edward H.

Clarke to the chair of Materia Medica. A Professorship of Clinical Medicine has also been created, and filled by Dr. George C. Shattuck, of Boston. Dr. E. Deming, Prof. of Pathology and Clinical Medicine in the Medical Department of the University of Missouri, died at his residence in Lafayette, Indiana, on the the 23d February, aged 58 years. Dr. Quintard has resigned the chair of Physiology and Pathology in the Memphis Medical College, and Dr. Rene La Roche of Philadelphia has been appointed to succeed him. The late class in the Memphis College presented Dr. Quintard, on his retirement, an elegant copy of the Bible as a token of their respect. We infer, from the comments of the Memphis Recorder, that the retiring Professor intends to enter the Ministry.

*To Our Subscribers.*—The present number makes the fourth of the present volume, and completes *one-third* of the present subscription year. Each number has been issued with regularity and promptness during the first week in each month. But not *one sixth* of our subscribers have yet forwarded the trifling amount which constitutes their individual subscriptions. Our Journal has a wide circulation, and if its subscribers pay up promptly, they will not only ensure our best possible exertions to make it interesting and valuable, but at the end of the present volume we shall put it in new type, and add sixteen pages to its reading matter. What say you, brethren of the profession in the North-west—shall we make our Journal not only one of the *best*, but also one of the largest in the Union? If you desire this, do your part, and I certainly will do *mine*. N. S. D.

*To Contributors.*—Communications have been received from Drs. Hunt, Higgins of Aurora, and Dodson of Morris. The first was furnished by the Secretary of the Æsculapian Society. All these will receive due attention in our next. Dr. Hutchinson is informed that his observations with the microscope would be highly acceptable. Indeed we wish many more of our patrons would favor us with the results of their experience and observations.

*College Vacancies.*—Notice is hereby given that, in consequence of the resignation of Prof. John McLean, the chair of Materia Medica and Therapeutics in Rush Medical College has become vacant. The chair of Physiology and Pathology in the same institution is also vacant. Applications for appointment to both these places will be received by the President, Prof. Daniel Brainard, until the 15th of June next.

*Health Officers.*—Dr. Isaiah P. Linn has been appointed City Physician for the ensuing year. Dr. B. McVickar is a member of the Board of Health. The Mayor, Dr. L. D. Boone, is also *ex-officio* member of the same board. The city of Chicago thus has the services of three medical men combining both talent and experience in her health department. The citizens will be justly disappointed unless better sanitary regulations are enforced than heretofore.

*Total Abstinence and Phthisis.*

A writer in the Boston Medical and Surgical Journal for Feb. 15th, 1855, over the signature of "Michigan," makes the following assertion :

"But the proposition is incontrovertible that *phthisis* has *increased* upon the spread of total abstinence in almost exact proportion."

Will our brother editors of the Boston Journal ask their correspondent, "Michigan," to give us and the world facts and figures on which he bases this "*incontrovertible proposition?*" They would be of much value to the profession, provided they have any existence. The same writer also says : "Alcohol, in its various forms, may be called upon through its easily-absorbed hydro-carbon to yield *heat and respiratory food.*"

Will he furnish the proof that alcohol, in *any* of its forms, is capable of yielding either?

*Fusel Oil in the Treatment of Phthisis.*

We find in the Boston Medical Journal for March 1st, two cases of well-marked tubercular phthisis reported, which were treated by fusel oil in the Massachusetts General Hospital. One patient took the oil seven months, during which time she gained 20 pounds in weight, and was discharged relieved. The other was under treatment with the oil eight months, during which time she gained in weight 13 pounds, and was much relieved when discharged. The oil was given in doses of from *four* to *six* drops after each meal. The fusel oil is obtained in the process of manufacturing alcohol from the more dilute spirits. It would seem to be worthy of further trial.

*Cubebs in Incontinence of Urine in Infants.*

The Memphis Medical Recorder says that "Dr. Driters believes that nocturnal incontinence in children is due to *atony* of the neck of the bladder, in which no remedy has greater power than *cubebs*." It should be given two or three times a day for some time. We have seldom failed to remedy the same class of cases by giving tinct. cantharides, in suitable doses, for a very few days. There are cases, however, dependent on *morbid sensitiveness* of the coats of the bladder, instead of *atony*, in which soothing and alkaline remedies are required.

*Fissures and Excoriations of the Nipple.*

M. Bourdel, of Montpelier, states, in the Gazette des Hospitome, that the tinct. of benzoin, when applied properly, is a sure remedy tor that most troublesome and painful affection termed fissures and excoriations of the nipples in nursing women. He applies the tincture with a hair pencil directly to the cracked or ulcerated surface, so as to completely cover it. The application should be repeated often enough to keep the covering that is left by the evaporation of the tincture complete. The first application produces considerable smarting, but the subsequent ones are painless, or nearly so; and the child continues to nurse, as though no application had been made. The cure is said to be effected in from one to two weeks.

*Orchitis, or Swelled Testicle.*

Several cases of this disease are related by Prof. Costes, *successfully* treated by keeping the scrotum covered with a mixture of twenty parts of collodion and six parts of castor oil. Dr. Ogier, of Charleston, S. C., in an article in the Charleston Medical Journal, recommends for the same disease, compression by means of an *oval bag*, made of thin India rubber thread, netted coarsely, and about the size of a turkey's egg.

This may be so stretched as to enclose the scrotum, and by its elasticity produces sufficient compression, without the trouble of removal and re-adjustment, as is the case with adhesive straps and bandages.

*Intense Cold as an Anæsthetic.*

Dr. J. C. Warren, of Boston, in a communication to the Boston Med. and Surg. Journal, adds his testimony in favor of the efficacy of intense cold, produced by a freezing mixture, in preventing pain during surgical operation. The method he adopts is the same as that recommended by Dr. Arnott, and is chiefly applicable to cases in which the parts to be operated on are superficial.

*Hospital for Females.*—Steps have been taken to organize and establish an Hospital in the City of New York, for the special treatment of the diseases of women. It is stated that Dr. J. Marion Sims is to be the attending Surgeon.

